# Worldwide clinical practices in perioperative antibiotic therapy for lung transplantation

**DOI:** 10.1186/s12890-020-1151-9

**Published:** 2020-04-29

**Authors:** Benjamin Coiffard, Eloi Prud’Homme, Sami Hraiech, Nadim Cassir, Jérôme Le Pavec, Romain Kessler, Federica Meloni, Marc Leone, Pascal Alexandre Thomas, Martine Reynaud-Gaubert, Laurent Papazian

**Affiliations:** 1 Department of Respiratory Medicine and Lung Transplantation, Aix Marseille University, APHM, Hôpital Nord, 13015 Marseille, France; 2 Aix Marseille University, APHM, Hôpital Nord, Intensive Care Unit, Marseille, France; 3 Aix Marseille University, IRD, APHM, MEPHI, IHU-Méditerranée Infection, Marseille, France; 4 Department of Cardio-Thoracic Surgery and Heart-Lung Transplantation, Hôpital Marie-Lannelongue, Le Plessis-Robinson, France; 5 Department of Respiratory Medicine and Lung Transplantation, Federation of Translational Medicine of Strasbourg (FMTS), Nouvel Hôpital Civil, Strasbourg, France; 6 Department of Medical Sciences and Infective Diseases, Unit of Respiratory Diseases, IRCCS Policlinico San Matteo Foundation, Pavia, Italy; 7 Aix Marseille University, APHM, Hôpital Nord, Department of Thoracic Surgery, Marseille, France; 8 Aix Marseille University, APHM, Hôpital Nord, Department of Anesthesiology, Marseille, France

**Keywords:** Survey, Lung transplantation, Antibiotic therapy, Perioperative, Bronchial colonization

## Abstract

**Background:**

Infection is the most common cause of mortality within the first year after lung transplantation (LTx). The management of perioperative antibiotic therapy is a major issue, but little is known about worldwide practices.

**Methods:**

We sent by email a survey dealing with 5 daily clinical vignettes concerning perioperative antibiotic therapy to 180 LTx centers around the world. The invitation and a weekly reminder were sent to lung transplant specialists for a single consensus answer per center during a 3-month period.

**Results:**

We received a total of 99 responses from 24 countries, mostly from Western Europe (*n* = 46) and the USA (*n* = 34). Systematic screening for bronchial recipient colonization before LTx was mostly performed with sputum samples (72%), regardless of the underlying lung disease. In recipients without colonization, antibiotics with activity against gram-negative bacteria resistant strains (piperacillin / tazobactam, cefepime, ceftazidime, carbapenems) were reported in 72% of the centers, and antibiotics with activity against methicillin-resistant *Staphylococcus aureus* (mainly vancomycin) were reported in 38% of the centers. For these recipients, the duration of antibiotics reported was 7 days (33%) or less (26%) or stopped when cultures of donor and recipients were reported negatives (12%). In recipients with previous colonization, antibiotics were adapted to the susceptibility of the most resistant strain and given for at least 14 days (67%).

**Conclusion:**

Practices vary widely around the world, but resistant bacterial strains are mostly targeted even if no colonization occurs. The antibiotic duration reported was longer for colonized recipients.

## Background

Infection is a significant complication following lung transplantation and represents the most common cause of mortality within the first year, but it is also a risk factor for chronic lung allograft dysfunction (CLAD) [1, 2]. Early infections are typically hospital-acquired, and more than half are bacterial pneumonia and surgical site infections [3]. Lung transplant recipients routinely receive perioperative antibiotic therapy, but antibiotic regimens vary widely depending on the underlying lung disease, pre-transplantation bacterial colonization, antibiotic susceptibility results and local protocols.

Clinical practice guidelines for antimicrobial prophylaxis in surgery recommend the use of cefazolin for heart and lung transplantations, but the evidence was mostly based on cardiac procedures [4]. Cystic fibrosis, chronic obstructive pulmonary disease (COPD) and less frequently interstitial lung diseases may have bronchial colonization by hospital-acquired microorganisms that possibly have multidrug resistance [5, 6]. Moreover, the emergence of pan-resistant organisms may occur, which is a relative contraindication for lung transplantation [7, 8]. In addition, donors are exposed to ventilator-associated pneumonia, which warrants consideration.

The management of antibiotics in such a context could be difficult, and strategies for decontamination and prolonged combination therapy are emerging [9]. Inversely, extended durations of broad-spectrum antimicrobials are a well-known risk factor for multidrug-resistant (MDR) bacterial and *Clostridioides difficile* infections [10–12]. There are currently no formal recommendations to guide antimicrobial selection in this specific context of lung transplant surgery [13]. Furthermore, there is also a lack of data concerning specific pathogens such as *Mycobacterium* and *Burkholderia* species [14–16].

The aim of this study was to assess the actual international practices of antibiotic therapy management carried out in the perioperative period of LTx.

## Methods

We used Google® and websites of the international organizations of LTx (ISHLT, UNOS, and Eurotransplant) to find all the centers that perform LTx around the world and physicians (and their emails) associated with these programs. We then used PubMed® to complete our mailing list with the MeSH terms “Lung Transplantation” followed by the “name of the physician(s)”, and/or “Hospital”, “City”, “Country” of the targeted centers. Missing emails were found through publications and information regarding the corresponding authors. The maximum email addresses for each LTx center was sought, and a total of 506 valid emails were collected from 180 centers in 35 countries.

The survey was developed by our transplant team (Marseille, France) between June and September 2018. During October 2018, the survey was sent to two other French LTx centers for reviewing and validation (Strasbourg and Le Plessis Robinson). In November 2018, the survey was sent to the mailing list with a personal link to an Internet service provider (https://docs.google.com/forms/). Only one response per center was requested. The answers were collected from November 2018 to January 2019. A reminder was sent every 15 days for 2 months and then weekly for the last month.

We designed a survey consisting of five short clinical vignettes potentially encountered in daily LTx practice, inquiring about local practices concerning the management of antibiotic therapy in the perioperative period of LTx. We considered the perioperative period as the period of the transplant surgery (per operative) and the post-surgery time before any infection occurrence (postoperative). After general questions on local practices, we asked each center for their diagnostic methods for microbial screening in recipients and donors. The clinical cases were related to specific issues concerning the management of antibiotic therapy in different clinical situations, including no prior colonization, prior colonization with MDR microorganisms (defined as non-susceptibility to at least 1 agent in 3 or more antimicrobial categories) [17], colonization definition, local tuberculoid granuloma in the lung explant, and prior colonization with MDR bacteria not susceptible to beta-lactams (complete survey in Additional file 1, raw answer data in Additional file 2). We hypothesized that, first, practices vary widely according to centers, and second, that antibiotic choices and duration of use may be different according to prior colonization, lung underlying diseases, and the profiles of sensitivity of the isolated bacteria in pre-transplant.

Analyses and graphical illustrations were performed with the public software R version 3.5.1 (R Core Team (2018). R: A language and environment for statistical computing. R Foundation for Statistical Computing, Vienna, Austria).

## Results

### General questions

**Fig. 1:**
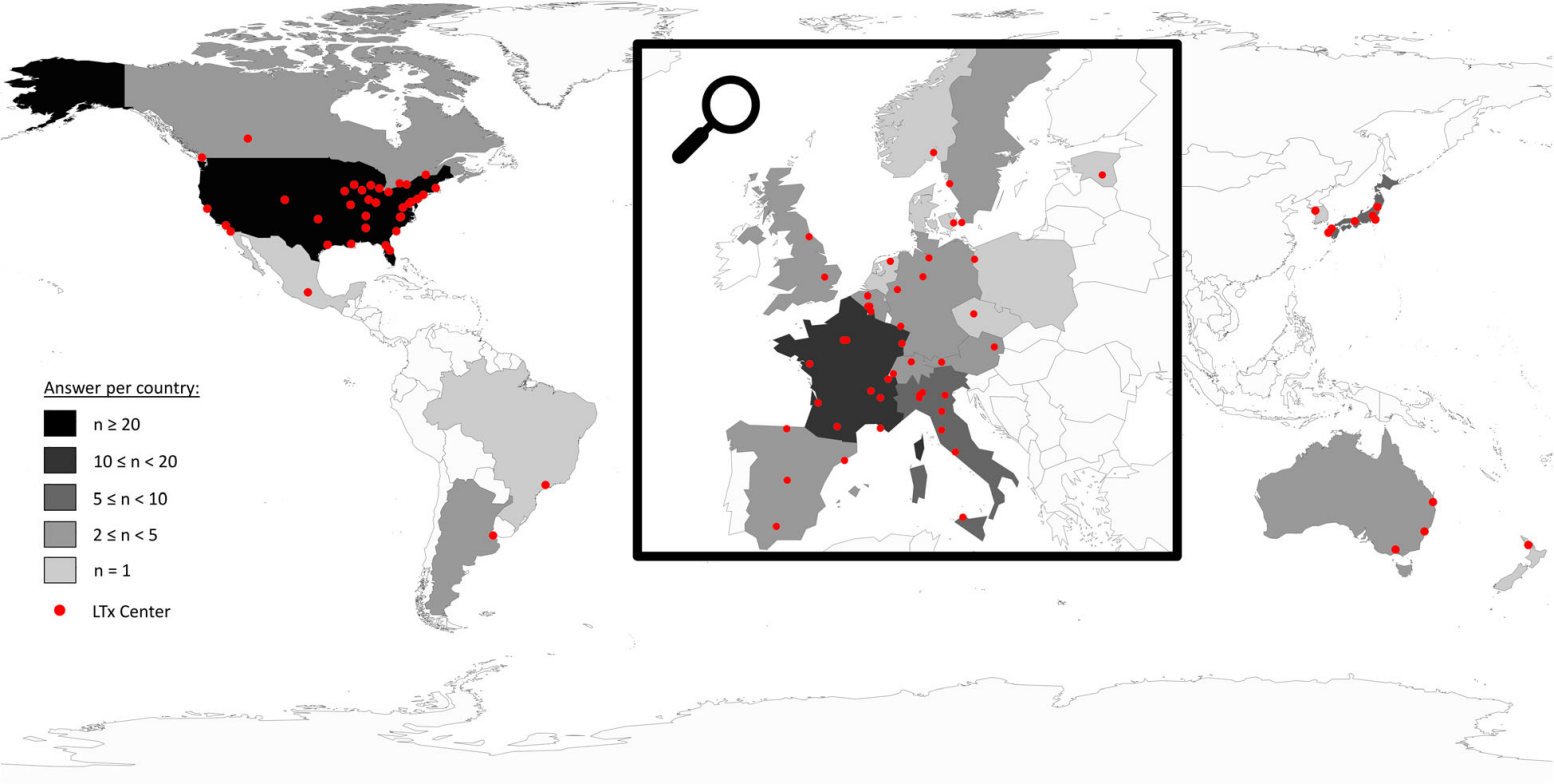
World map representing the lung transplant centers answering the survey and the number of responses by country. The map was generated with the public R software using the “maps” package

**Fig. 2:**
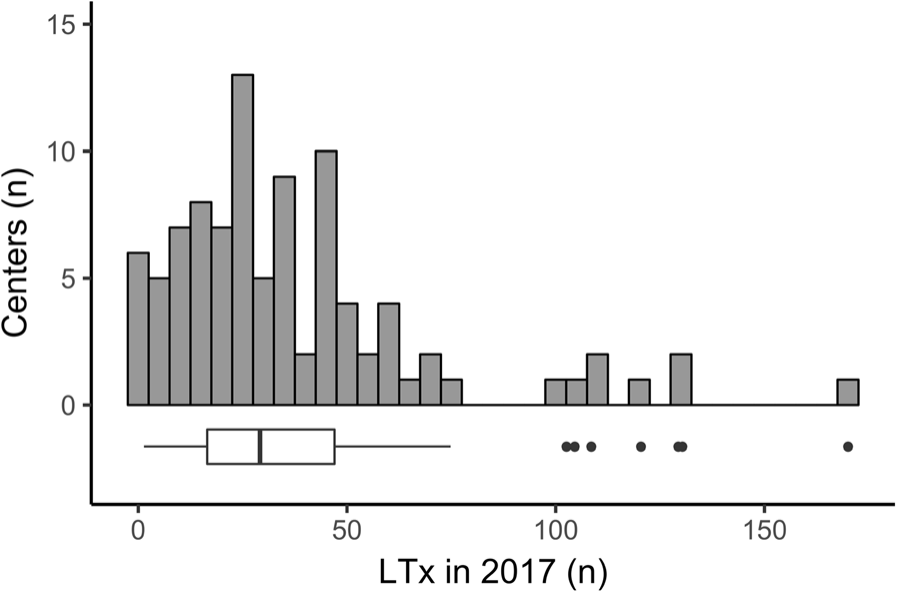
Histogram and Boxplot representing the distribution of the number of lung transplantations per center performed in 2017. The bars are per slice of 5 lung transplantations. The boxplot corresponds to the median with the interquartile range (distance between the first and third quartiles); the lower and upper whiskers extend from the hinge to the lowest and highest (respectively) values that are within 1.5 x IQR of the hinge LTx: lung transplantation.

We received responses from 99 centers (55% return rate), which represent more than 3617 LTx performed in 2017. Answers came from 24 different countries (Fig. 1), mostly from Western Europe (*n* = 46) and the USA (*n* = 34). The median [interquartile] number of LTx per center was 28 (15–38) in 2017 (Fig. 2). Details concerning general questions on LTx performed in each center are reported in Table 1. Of the centers, 68.7% reported having a protocol for the management of perioperative antibiotic therapy.

**Table 1:** Answers to the questions concerning the general practice of lung transplantation for each center

Question	Answer	n	%
What is your specialty in the lung transplant program?			
Pulmonologist		69	69.7
Surgeon		18	18.2
Infectious disease physician		7	7.1
Intensivist		3	3.0
Anesthesiologist		1	1.0
Internist		1	1.0
Nurse Practitioner		1	1.0
No answer		0	0.0
*Who is in charge of the antibiotic prophylaxis management?*			
Pulmonologist		69	69.7
Infectious disease physician		21	21.2
Surgeon		8	8.1
Multi-disciplinary		5	5.1
Intensivist		2	2.0
Anesthesiologist		1	1.0
Internist		1	1.0
No answer		0	0.0
What is the main indication for lung transplant in your program?			
ILD		60	60.6
COPD		26	26.3
CF		19	19.2
Mixte		10	10.1
PH		4	4.0
No answer		0	0.0
Do you perform a specific induction therapy?			
Anti-IL2R		56	56.6
Steroids only		27	27.3
ATG		24	24.2
No induction		9	9.1
Alemtuzumab		7	7.1
No answer		1	1.0
What is the post-transplant recipient location?			
Cardiothoracic ICU		56	56.6
Medical-Surgical ICU		19	19.2
Surgical ICU		13	13.1
Transplant ICU		7	7.1
Medical ICU		4	4.0
No answer		0	0.0

*ILD* Interstitial lung disease, *COPD* Chronic obstructive pulmonary disease, *CF*CYSTIC fibrosis, *PH* Pulmonary hypertension, *Anti-IL2R* Anti-IL2 receptor (basiliximab or daclizumab), *ATG* Anti-thymocyte globulins, *ICU* Intensive care unit

#### Case 1: no prior colonization (Additional file 3)

The first case was a LTx with no known bronchial colonization. Systematic screening for bronchial colonization before LTx was performed mainly with sputum samples regardless of the underlying lung disease.

**Fig. 3:**
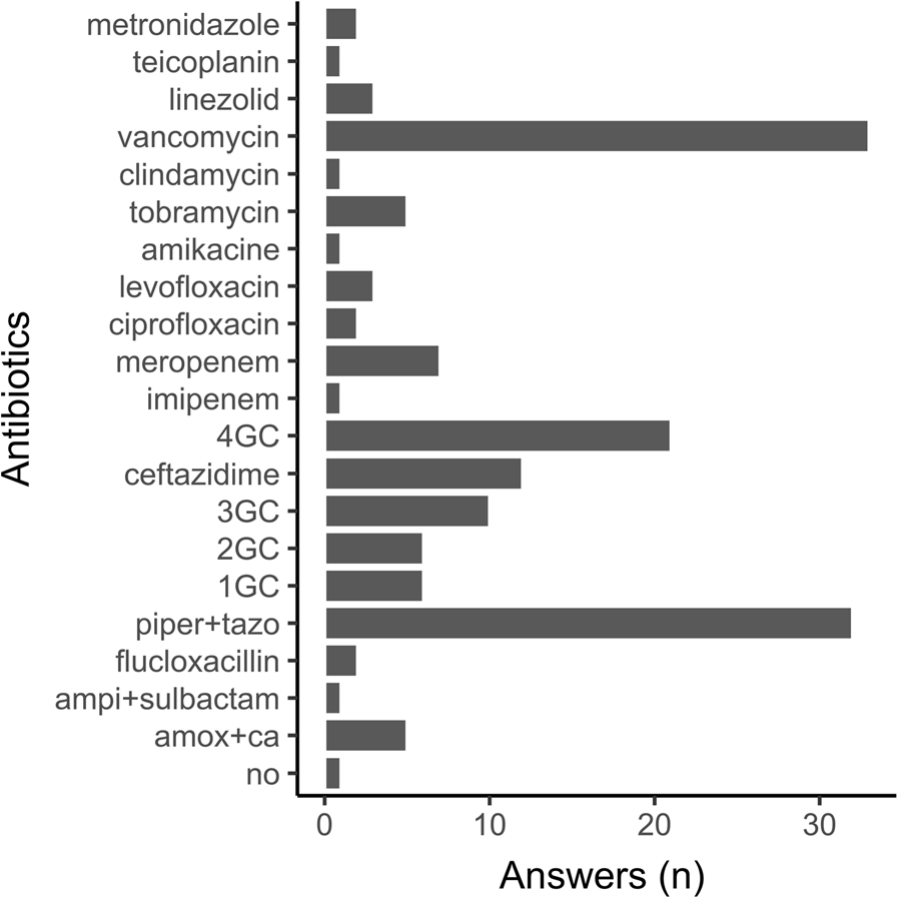
Bar plot representing the number of responses per antibiotic for Case 1 about antibiotic prophylaxis for interstitial lung disease without bronchial colonization. Amox+ca: amoxicillin+clavulanic acid; ampi+sulbactam: ampicillin+sulbactam; piper+tazo: piperacillin+tazobactam; 1GC: first-generation cephalosporins; 2GC: second-generation cephalosporins; 3GC: third-generation cephalosporins; 4GC: fourth-generation cephalosporins

The most commonly used antibiotics were piperacillin/tazobactam (32.3%), fourth-generation cephalosporins such as cefepime (21.2%), and antibiotics with activity against methicillin-resistant *Staphylococcus aureus* (MRSA) (37.7%), mostly vancomycin (86%) (Fig. 3). Antibiotics with activity against MRSA were almost exclusively used by centers from the USA (84%) and systematically associated with beta-lactam and preferentially (89%) beta-lactam with activity against gram-negative bacteria (GNB)-resistant strains (piperacillin/tazobactam, cefepime, ceftazidime or carbapenem).

**Fig. 4:**
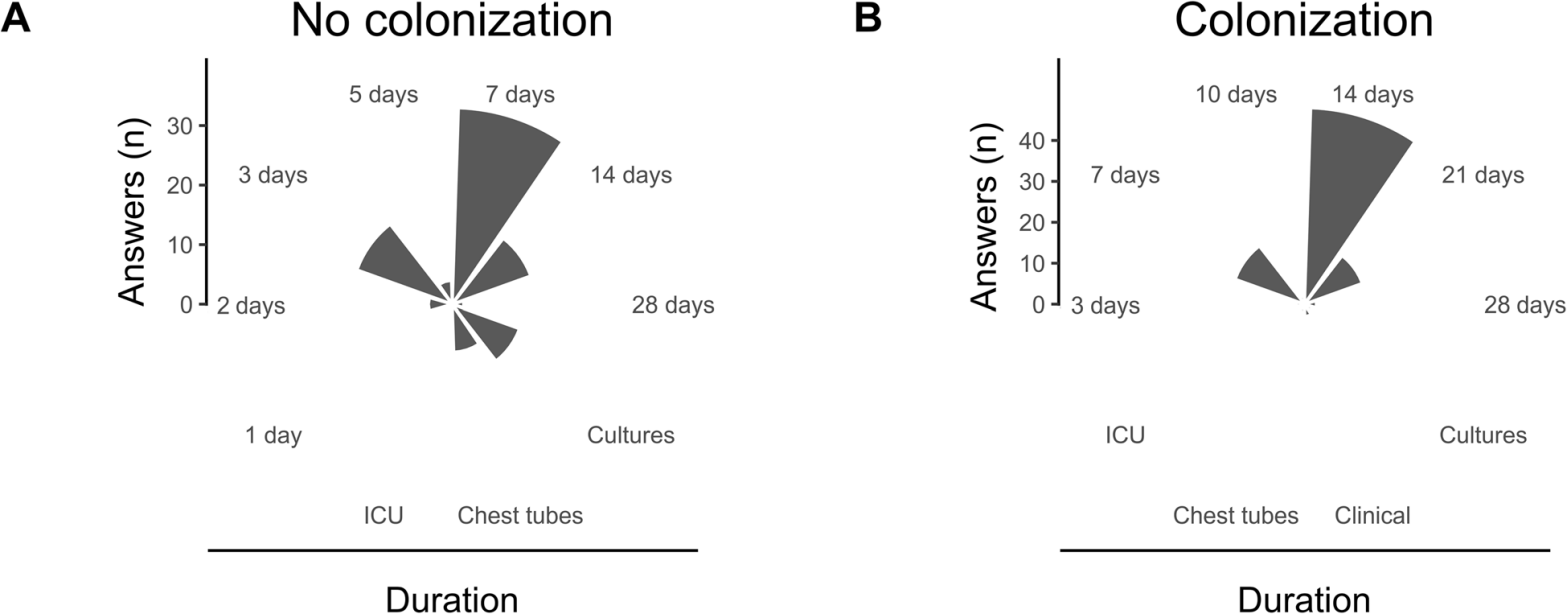
Polar bar plot representing the number of responses for the duration of antibiotic prophylaxis in the context of no colonization (**a**, Case 1) or colonization (**b**, Case 2). Cultures (plot **a**): until donor and recipient cultures are reported negatives; Cultures (plot **b**): according to donor and recipient cultures; Chest tubes: until indwelling chest tubes are removed; ICU: until ICU discharge; Clinical: according to clinical course

The duration of prophylaxis in this context was very heterogeneous (Fig. 4a) but mostly 7 days (33.3%) or shorter (26.3%), or until cultures of the donor and the recipients were reported as negatives (12.1%). The antibiotic treatment was almost systematically adapted to the results of the donor samples (97.1%). After 4 days of empiric treatment, if the results of the bacteriological screening were negative, and there was no sign of infection, antibiotics were stopped in 52.5% of the centers.

#### Case 2: prior MDR colonization (Additional file 4)

The second clinical case was a LTx for cystic fibrosis with colonization with *Pseudomonas aeruginosa* only susceptible to carbapenems, colistin and tobramycin. This colonization was not considered a contraindication in 94.9% of the centers, and there was no pretransplant decolonization strategy in 69.7%. The postoperative antimicrobial prophylaxis consisted of meropenem or imipenem (92.9%), tobramycin (45.5%), colistin (36.3%), and antibiotics with activity against MRSA pathogens such as vancomycin, linezolid or teicoplanin (25.3%). Combined antibiotics with a carbapenem (or a new antipseudomonal cephalosporin with a beta-lactamase inhibitor) and tobramycin or colistin were proposed in 69.7%. The duration of this antibiotic treatment was very heterogeneous (Fig. 4b) but was at least 14 days in 66.7% of the centers. After 4 days of antibiotic treatment, even if the results of the bacteriological samples issued from the donor and the recipient were negative and without any sign of infection, antibiotics were not stopped in 89.9% of the centers. Similarly, cases with colonization by a *Burkholderia species* were not considered absolute contraindications for LTx by 11.1% of the respondents unless the strain was pan-resistant (38.4%).

#### Case 3: definition of colonization (Additional file 5)

The third clinical case was the definition of colonization with wild-type or MDR bacteria in a COPD recipient. The questions were related to the delay between the last bacterial isolation and the LTx to consider if the therapy should target these bacteria. In such cases, where wild bacteria (*Pseudomonas aeruginosa*) were isolated at least once and never found again on the last samples, the delay was widely heterogeneous: 15 days or less (14.2%), between 1 and 3 months (35.3%), 6 months (16.2%), and 1 year or more (28.2%). If the organism was an MDR strain, the duration to consider antibiotic prophylaxis targeting this bacteria was longer: 15 days or less (14.2%), between 1 and 3 months (27.3%), 6 months (22.2%), and 1 year or more (34.3%).

#### Case 4: tuberculoid granuloma in the lung explant (Additional file 6)

The fourth case focused on the management of a localized tuberculoid granuloma with caseous necrosis found by pathology on the lung explant with no clinical or radiological evidence of active mycobacterial infection after transplantation. Concerning the strategy to identify a causal mycobacterial agent, 51.5% of the centers performed specific mycobacterial PCR on lung explant tissue, 58.6% performed a bronchoalveolar lavage in the recipient for culture, and 54.5% repeated the screening for mycobacterium species in the recipient. In such a context, 42.4% performed a specific treatment for latent tuberculosis.

#### Case 5: MDR colonization not sensitive to beta-lactams (Additional file 7**)**

The fifth case was a LTx for cystic fibrosis with a history of colonization by MRSA and MDR strains of *Pseudomonas aeruginosa* only sensitive to ciprofloxacin, amikacin and colistin. Postoperative therapy against MRSA was performed with vancomycin (62.6%) or linezolid (30.3%). An antipseudomonal beta-lactam associated with ciprofloxacin or amikacin was also used in 26.3% of the centers, ciprofloxacin associated with amikacin alone was used in 21.2% of the centers, and nebulized colistin was used in 35.4% of the centers regardless of the other antibiotics.

On day 5, if the organism retrieved in the recipient perioperative samples was a wild type *Pseudomonas aeruginosa,* with no clinical signs of infection, 60.6% of the centers did not perform de-escalation, and 17.2% proposed de-escalation with piperacillin + tazobactam.

### Practice according to large and small volume centers

We compared the practice on perioperative antibiotic therapy according to large and small volume centers (Additional file 8). The groups were defined on the median of lung transplants performed in 2017 (median = 28 LTx/year). Clinical practice on perioperative antibiotic therapy were similar between groups concerning microbiological screening, definition of a pre-transplant colonization, type and length of antimicrobial prophylaxis. Responders were more likely Pulmonologist in larger volume centers and more Surgeon in small volume centers (*p* = 0.02). A team protocol for antimicrobial prophylaxis was more reported in larger volume centers (80% versus 59%, p = 0.02). Pre-transplant *Burkholderia species* colonization was more considered as absolute contraindication in small volume centers and rather a contraindication only if pan-resistant in larger volume centers.

## Discussion

The responses to our survey showed highly variable practices regarding the use of antibiotics in the perioperative period of LTx. Despite American guidelines for antimicrobial prophylaxis in surgery that recommended the use of cefazolin for heart and lung transplantations [4] (mostly to prevent the risk of surgical site infections), most of the centers (> 70%) used antibiotics against GNB-resistant strains (piperacillin/tazobactam, cefepime, ceftazidime or carbapenem), even if no previous bronchial colonization had occurred. Several studies reported that *Pseudomonas aeruginosa* was the most frequent microorganism to cause infections following LTx*,* with an occurrence rate between 25 and 60% [18–21].

Half of the infectious episodes following LTx occur in the first 30 days [18] and are derived from the recipient and/or donor or are the consequence of the induction of immunosuppressive therapy and are mainly hospital-acquired. Dudau et al. demonstrated that severities of illness and lung injury were the two major risk factors for nosocomial pneumonia recurrence despite antimicrobial therapy [21]. Nevertheless, pretransplant colonization was described as the main predictive factor of developing an infection in the postoperative period [19, 20]. Regarding this aspect, the responses of the survey were quite similar, and all centers adjusted antibiotic therapies to previous colonization when present.

Donors are also potential sources of infection and donor-derived infections in lung transplant recipients have been reported in 5–20% of cases, some with fatal outcomes [22–25], and justify antibiotic therapy against GNB-resistant strains, especially since donor lung criteria were extended to marginal donors, including donors with bronchial secretions and with prolonged mechanical ventilation [26–28]. Antibiotics may allow a remarkable decrease in donor-derived infections (from 5.7 to 2.9%) in a single-center study after performing tailored antibiotic treatment even when these antibiotics are given as nebulized administration [25]. An old study in 37 recipients demonstrated that organisms isolated from the donor tracheal cultures were different from those associated with early infections [29]. In any case, donor-derived infections would be in the context of ventilator-associated pneumonia (VAP), and international guidelines recommend including coverage for *Staphylococcus aureus*, *Pseudomonas aeruginosa*, and other gram-negative bacilli in all empiric regimens of VAP [30]. For the risk of donor-derived infection, the answers were homogeneous since almost all centers adjusted their therapy according to the donor results.

The risk of *Staphylococcus aureus* may be multifactorial (from previous bronchial colonization, the donor, the skin incision for the surgery, or post-LTx VAP). Our survey revealed that only one-third of the centers target MRSA. Antibiotics against MRSA were almost exclusively used by centers from the USA, which follows the direction of use in accordance with the local ecology of each institution. Vancomycin was the most commonly used molecule against MRSA (> 80%). The perioperative period of a LTx may be associated with hemodynamic instability and carries a risk of acute kidney injury, but higher nephrotoxicity for vancomycin compared to linezolid was never clearly demonstrated [31]. However, it was demonstrated that linezolid has better penetration into the lung [32]. Thus, the preferential use of vancomycin could be historical but could also be for pharmacodynamic reasons since vancomycin may be administered continuously and easily monitored by blood dosage.

Colonization is usually described as the detection of at least two isolates of an organism separated by a certain amount of time [33]. In this specific context of end-stage chronic pulmonary disease at high risk of hospital-acquired infection and the unknown timing of transplant when listed, bronchial colonization risk is difficult to manage. Thus, the time to define colonization and the risk of the recurrence of an agent already being isolated is not clear. For this purpose, responses were clearly heterogeneous, and the delay between the last bacteria isolation and the LTx for considering targeting the bacteria varied from 15 days until more than 1 year. Interestingly, there was a trend to consider a longer delay to define colonization with MDR bacteria.

Despite the risk of bronchial colonization, only one-third of the centers reported a pretransplant decolonization strategy. Candidates for a LTx with bronchial colonization have recurrent episodes of infection in their history despite repeated antibiotic cures. Thus, classical antibiotic strategies, such as parenteral or nebulized antibiotics, are probably considered ineffective. However, alternative decolonization strategies are emerging in the perioperative period of a LTx. Indeed, different solutions have been tested with interesting results, such as perioperative tracheobronchial lavage and/or pleural irrigation with antiseptic solutions [34, 35] or a combination of antibiotic therapies [36, 37].

Multidrug-resistant bacteria are a specific concern in lung transplantation. We observed different antibiotic strategies between non-colonization recipients and recipients colonized with MDR bacteria in our survey, even under similar clinical conditions. Indeed, the presence of MDR bacteria followed the direction of the highest “caution” and, as an example, longer antibiotic therapy and less de-escalation were the reported responses despite a well-known risk factor for multidrug-resistant (MDR) bacterial and *Clostridioides difficile* infections in longer therapy [10–12]. It is still debated whether MDR bacteria are more problematic than wild-type agents. Recently, a study from the ISHLT registry demonstrated a similar survival rate in cystic fibrosis lung transplant recipients infected with organisms labeled pan-resistant despite more infection episodes [8]. Although, in this study, there are no precise data about the bacterial species or the antibiotic treatments, most of the centers did not consider MDR bacteria a contraindication to transplantation, except in the context of pan-resistance, which is still considered at higher risk. For some species like *Burkholderia cenocepacia* many studies reported an increased mortality after LTx and for that reason, this colonization has been considered a contraindication in some centers [38–40]. Aguado et al. [41] recently recommended that MDR bacteria should not constitute a contraindication to transplantation but highlighted the importance of characterizing the isolate’s phenotypic and genotypic resistance profile to better guide treatment.

Risk management of *Mycobacterium tuberculosis* is also not consensual. Less than half of the centers reported a systematic screening and a specific treatment when positive for latent tuberculosis in the pretransplant evaluation. Lung transplant recipients are at higher risk of tuberculosis, and recently, guidelines recommended systematic screening and treatment for latent tuberculosis in the pretransplant assessment [42].

Antibiotic use is a well-known risk factor for MDR and *Clostridioides difficile* infections. The right balance to prevent infection risk with limited side effects is not known. The duration of gram-positive antimicrobials has been described to increase the risk of MDR and *Clostridioides difficile* infection in an analysis of 500 non-cystic fibrosis lung transplant patients [10]. In our survey, some centers adapted and stopped antibiotic therapy as soon as possible if the results of the bacteriological screening in donor and recipient were negative and there was no sign of infection. Conversely, there was a trend toward a longer duration of antibiotic therapy in the context of cystic fibrosis and MDR agents.

Our study has several limitations. Some respondents (mainly surgeons) were not in charge of antibiotic management, and thus, in those cases, the answers might maybe not reflect the real practice of these centers. The question of the survey is complex and the practice varies widely from one center to another. Thus it was really challenging to cover all aspects of the problematic and in order not to make the questions too long, we sometimes limited the choice of answers and certain situations may have been neglected. For instance, the route of administration was not always specified when asking for an antibiotic strategy. Moreover, not all 180 centers contacted have responded to the survey, but we received a global response rate of 55%. The response rate from Western Europe was the highest (62%), but some countries with experience in LTx, such as the United Kingdom and Germany, did not participate well in the survey (33 and 27%, respectively). We received no answer from China. Because we did not screen centers in advance for eligibility, it is likely that some of the nonresponders would have been ineligible (because they do not perform LTx). Furthermore, some centers may be not used to complex infectious situations since most centers perform LTx for fibrosis (61%) and may not be used to cystic fibrosis, which entails a more complex risk.

Our study revealed high heterogeneity between centers. It could be interesting now to evaluate in further appropriate randomized studies the use of antibiotics in perioperative of a LTx. From our point of view, two major questions still remain to be evaluated. Firstly, the type and the spectrum of the antibiotic therapy used, particularly in no prior colonization. And second, the duration of the postoperative antibiotic use on the prevention of postoperative infections on one hand, and on the selection of MDR strains or the occurrence of *Clostridioides difficile* infections on the other hand.

## Conclusion

These survey results suggest that practices vary widely around the world, but hospital-acquired bacteria are mostly targeted by perioperative antibiotic therapy even if no colonization occurs, probably in order to treat possible donor-derived infections. Furthermore, there is a trend to limit antibiotic duration if the results of the bacteriological screening in the donor and recipient are negative and show no sign of infection, but the duration reported was longer for colonized recipients, cystic fibrosis patients and MDR situations.

## Supplementary information

**Additional file 1.** Survey sent to the lung transplantation centers.

**Additional file 2.** Raw anonymized answers of the 99 participants

**Additional file 3.** Answers to Case 1.

**Additional file 4.** Answers to Case 2.

**Additional file 5.** Answers to Case 3.

**Additional file 6.** Answers to Case 4.

**Additional file 7.** Answers to Case 5.

**Additional file 8 **Answers according to large et small volume centers. The groups were defined on the median of lung transplants performed in 2017 (*n* = 28). LTx: lung transplantation.

## Data Availability

The complete survey and all anonymized data are available in Supplementary materials (Additional files 1 and 2).

## Abbreviations

CLAD: Chronic lung allograft dysfunction
COPD: Chronic obstructive pulmonary disease
GNB: Gram-negative bacteria
LTx: Lung transplantation
MDR: Multidrug-resistant
MRSA: Methicillin-resistant *Staphylococcus aureus*
VAP: Ventilator-associated pneumonia
