# Supplementary material for: Worldwide clinical practices in perioperative antibiotic therapy for lung transplantation
Source: BMC Pulm Med. 2020 Apr 29;20:109. doi: 10.1186/s12890-020-1151-9 (PMC7191774; doi:10.1186/s12890-020-1151-9)
Supplement: Supplementary file 1 — Additional file 1. Survey sent to the lung transplantation centers. [file 12890_2020_1151_MOESM1_ESM.docx]

**Supplementary Text S1: Survey sent to the lung transplantation centers.**

Object: International survey on antibiotic prophylaxis in lung transplantation

Dear Professors, Dear Colleagues,

As you already know, infection is a significant complication following lung transplantation, representing the most common cause of mortality within the first year. Early infections are typically nosocomial and more than half of these are bacterial pneumonia and surgical site infections. Lung transplant recipients routinely receive postoperative antimicrobial prophylaxis in addition to perioperative prophylaxis. Because of the lack of international guidelines, antibiotic regimens vary widely depending on the underlying lung disease, pre-transplantation bacterial status, antibiotic susceptibility results and local protocols.

The aim of this study is to assess the antibiotic prophylaxis management carried out in the peri-operative period following lung transplantation. This survey is a multicenter international study sent to 174 centers around the world.

The questions will deal with short clinical cases, potentially encountered in daily lung transplantation practice, and will only take few minutes of your precious time. Please, answer to these questions according to your clinical routine practice.

We need one answer per center. If you are not the referent person concerning this topic, we would be grateful to you to transfer this survey to this person.

If you accept to participate to this survey, you will be quoted as a collaborator in case of publication.

We thank you for reading this email and wish you the best for you, your team and your patients.

Best regards,

Benjamin COIFFARD, MD

Intensive Care Unit, Lung Transplant Center, Hôpital Nord, Marseille, France

benjamin.coiffard@ap-hm.fr

Eloi PRUD'HOMME, MD

Intensive Care Unit, Lung Transplant Center, Hôpital Nord, Marseille, France

Nadim CASSIR, MD, PhD

Infectious Diseases and Infection Control, Lung Transplant Center, Hôpital Nord, Marseille, France

Sami HRAIECH, MD, PhD

Intensive Care Unit, Lung Transplant Center, Hôpital Nord, Marseille, France

Martine REYNAUD-GAUBERT, MD, PhD

Respiratory Diseases, Lung Transplant Center, Hôpital Nord, Marseille, France

Pascal Alexandre THOMAS, MD, PhD

Thoracic Surgery, Lung Transplant Center, Hôpital Nord, Marseille, France

Laurent PAPAZIAN, MD, PhD

Intensive Care Unit, Lung Transplant Center, Hôpital Nord, Marseille, France

**General questions**

**What country are you from?**

**What city are you from?**

**What is the name of your Hospital?**

**What is your specialty in the lung transplant program?**

**General questions about your practice**

**How many lung transplant procedures were performed in 2017 in your center (uni or bilateral lungs, heart-lung procedure)?**

**Do you perform a specific induction therapy?**

**(multiple choices possible)**

1. no induction (neither corticosteroids or others)
2. corticosteroids only
3. anti-lymphocytes globulins
4. anti-thymocytes globulins
5. monoclonal IL2 receptor antagonist (basiliximab, daclizumab)
6. alemtuzumab
7. other

**What is the main indication for lung transplant in your program?**

**(multiple choices possible)**

1. COPD
2. interstitial lung diseases
3. cystic fibrosis
4. pulmonary hypertension
5. no predominant pathology
6. other

**What is the post-transplant recipient location?**

**(one choice possible)**

1. Cardiothoracic surgical ICU
2. Surgical ICU
3. Medical-surgical ICU
4. Medical ICU
5. Transplant ICU
6. Other

**Who is in charge of the antibiotic prophylaxis management?**

**(one choice possible)**

1. Intensivist
2. Pulmonologist
3. Surgeon
4. Anesthesiologist
5. Infectiologist
6. Other

**Case 1**

**A 56-year-old man will benefit from a lung transplantation in your center for end-stage interstitial lung disease. He has no known bronchial colonization in his medical history.**

**In your pretransplantation assessment, do you usually perform a systematic screening with microbial tests for patients with INTERSTITIAL LUNG DISEASE?**

**(multiple choices possible)**

1. never
2. sputum sample(s)
3. tracheal aspirate(s)
4. endoscopic bronchial aspiration
5. broncho-alveolar lavage(s)
6. depending on the arterial blood gases
7. tuberculosis blood test (Interferon-γ release assays testing, IGRA)
8. other

**If the patient had a COPD rather than interstitial lung disease would you perform systematic screening?**

**(multiple choices possible)**

1. no sample
2. same as interstitial lung disease
3. sample systematically performed unlike in interstitial lung disease
4. sputum sample(s)
5. tracheal aspirate(s)
6. endoscopic bronchial aspiration
7. broncho-alveolar lavage(s)
8. depending on the arterial blood gases
9. other

**If the patient had a CYSTIC FIBROSIS rather than interstitial lung disease would you perform systematic screening?**

**(multiple choices possible)**

1. no sample
2. same as interstitial lung disease
3. sample systematically performed unlike in interstitial lung disease
4. sputum sample(s)
5. tracheal aspirate(s)
6. endoscopic bronchial aspiration
7. broncho-alveolar lavage(s)
8. depending on the arterial blood gases
9. other

**The patient has no bronchial colonization. Which PERIOPERATIVE antimicrobial prophylaxis would you provide during the lung transplant surgery?**

**(multiple choices possible)**

1. no prophylaxis
2. amoxicillin
3. amoxicillin + clavulanic acid
4. first-generation cephalosporin (e.g. cefazolin)
5. second-generation cephalosporin (e.g. cefuroxime)
6. third-generation cephalosporins (e.g. cefotaxime or ceftriaxone)
7. antipseudomonal third-generation cephalosporins (e.g. ceftazidime)
8. fourth-generation cephalosporins (e.g. cefepime)
9. fifth-generation cephalosporins (e.g. ceftaroline)
10. piperacillin + tazobactam
11. carbapenem: imipenem
12. carbapenem: meropenem
13. carbapenam: doripenem
14. antibiotics with activity against MRSA pathogens: vancomycin
15. antibiotics with activity against MRSA pathogens: linezolid
16. antibiotics with activity against MRSA pathogens: teicoplanin
17. fluoroquinolone: ciprofloxacin
18. fluoroquinolone: levofloxacin
19. aminoglycoside: amikacin
20. aminoglycoside: tobramycin
21. aminoglycoside: gentamycin
22. colimycin
23. other

**The lung transplantation was successfully performed and the recipient transferred to the intensive care unit. Which POSTOPERATIVE antimicrobial prophylaxis would you provide during the postoperative period for this patient?**

**(multiple choices possible)**

1. no prophylaxis
2. amoxicillin
3. amoxicillin + clavulanic acid
4. first-generation cephalosporin (e.g. cefazolin)
5. second-generation cephalosporin (e.g. cefuroxime)
6. third-generation cephalosporins (e.g. cefotaxime or ceftriaxone)
7. antipseudomonal third-generation cephalosporins (e.g. ceftazidime)
8. fourth-generation cephalosporins (e.g. cefepime)
9. fifth-generation cephalosporins (e.g. ceftaroline)
10. piperacillin + tazobactam
11. carbapenem: imipenem
12. carbapenem: meropenem
13. carbapenam: doripenem
14. antibiotics with activity against MRSA pathogens: vancomycin
15. antibiotics with activity against MRSA pathogens: linezolid
16. antibiotics with activity against MRSA pathogens: teicoplanin
17. fluoroquinolone: ciprofloxacin
18. fluoroquinolone: levofloxacin
19. aminoglycoside: amikacin
20. aminoglycoside: tobramycin
21. aminoglycoside: gentamycin
22. colymicin (IV and/or aerosol)
23. other

**What DURATION of postoperative antimicrobial prophylaxis would you choose in this patient?**

**(one choice possible)**

1. no prophylaxis
2. 1 day
3. 3 days
4. 7 days
5. 14 days
6. 21 days
7. 28 days
8. until indwelling chest tubes are removed
9. until ICU discharge
10. other

**In case of positive Interferon-γ release assays for tuberculosis before lung transplantation, do you treat this latent tuberculosis in the absence of previous and appropriate treatment?**

**(one choice possible)**

1. No
2. Yes, before lung transplantation
3. Yes, after lung transplantation
4. Yes, before AND after lung transplantation
5. other

**In routine, do you have the results of bacteriological samples issued from the donor? If yes, what kind?**

**(multiple choices possible)**

1. no
2. preserving liquid
3. bronchial cut
4. tracheal aspirate or BAL before donor lung explant
5. tracheal aspirate or BAL during lung transplant surgery
6. other

**Do you adapt your antibiotic prophylaxis according to the results of the donor samples?**

**(one choice possible)**

1. yes
2. no

**At day 4 following lung transplantation, lung bacteriological samples issued from the donor and the recipient are sterile. The patient has no sign of infection. Would you stop antibiotic prophylaxis (if still prescribed)?**

**(one choice possible)**

1. Yes
2. No

**Case 2**

**A 28-year-old woman will benefit from a lung transplantation in your center for end-stage cystic fibrosis. She has bronchial colonization in her medical history since many years, with regular acute exacerbations (six last year). The last broncho-alveolar lavage performed 2 weeks ago confirms the presence of a multi-resistant Pseudomonas aeruginosa, only susceptible to carbapenems, colimycin and tobramycin.**

**Do you consider this colonization as a contraindication for lung transplantation?**

**(one choice possible)**

1. No
2. Yes, absolute contraindication
3. Yes, relative contraindication

**Apart from an episode of exacerbation, do you perform in this patient pre-transplant decolonization strategy?**

**(multiple choices possible)**

1. no
2. parenteral antibiotic therapy: 1 week
3. parenteral antibiotic therapy: 2 weeks
4. parenteral antibiotic therapy: more than 2 weeks
5. parenteral antibiotic therapy: until transplantation
6. aerosol antibiotic therapy: 1 week
7. aerosol antibiotic therapy: 2 weeks
8. aerosol antibiotic therapy: more than 2 weeks
9. aerosol antibiotic therapy: until transplantation
10. other

**Which PERIOPERATIVE antimicrobial prophylaxis would you prescribe for lung transplant surgery in this patient?**

**(multiple choices possible)**

1. no prophylaxis
2. amoxicillin
3. amoxicillin + clavulanic acid
4. first-generation cephalosporin (e.g. cefazolin)
5. second-generation cephalosporin (e.g. cefuroxime)
6. third-generation cephalosporins (e.g. cefotaxime or ceftriaxone)
7. antipseudomonal third-generation cephalosporins (e.g. ceftazidime)
8. fourth-generation cephalosporins (e.g. cefepime)
9. fifth-generation cephalosporins (e.g. ceftaroline)
10. piperacillin + tazobactam
11. carbapenem: imipenem
12. carbapenem: meropenem
13. carbapenam: doripenem
14. antibiotics with activity against MRSA pathogens: vancomycin
15. antibiotics with activity against MRSA pathogens: linezolid
16. antibiotics with activity against MRSA pathogens: teicoplanin
17. fluoroquinolone: ciprofloxacin
18. fluoroquinolone: levofloxacin
19. aminoglycoside: amikacin
20. aminoglycoside: tobramycin
21. aminoglycoside: gentamycin
22. colimycin
23. other

**The lung transplantation was successfully performed and the recipient transferred to the intensive care unit. Which POSTOPERATIVE antimicrobial prophylaxis would you provide during the postoperative period for this patient?**

**(multiple choices possible)**

1. no prophylaxis
2. amoxicillin
3. amoxicillin + clavulanic acid
4. first-generation cephalosporin (e.g. cefazolin)
5. second-generation cephalosporin (e.g. cefuroxime)
6. third-generation cephalosporins (e.g. cefotaxime or ceftriaxone)
7. antipseudomonal third-generation cephalosporins (e.g. ceftazidime)
8. fourth-generation cephalosporins (e.g. cefepime)
9. fifth-generation cephalosporins (e.g. ceftaroline)
10. piperacillin + tazobactam
11. carbapenem: imipenem
12. carbapenem: meropenem
13. carbapenam: doripenem
14. antibiotics with activity against MRSA pathogens: vancomycin
15. antibiotics with activity against MRSA pathogens: linezolid
16. antibiotics with activity against MRSA pathogens: teicoplanin
17. fluoroquinolone: ciprofloxacin
18. fluoroquinolone: levofloxacin
19. aminoglycoside: amikacin
20. aminoglycoside: tobramycin
21. aminoglycoside: gentamycin
22. colymicin (IV and/or aerosol)
23. other

**What DURATION of postoperative antimicrobial prophylaxis would you choose in this patient?**

**(one choice possible)**

1. no prophylaxis
2. 1 day
3. 3 days
4. 7 days
5. 14 days
6. 21 days
7. 28 days
8. until indwelling chest tubes are removed
9. until ICU discharge
10. other

**At day 4 following lung transplantation, lung bacteriological samples issued from the donor and the recipient are sterile. The patient has no sign of infection. Would you stop antibiotic prophylaxis (if still prescribed)?**

**(one choice possible)**

1. Yes
2. No

**If instead of the Pseudomonas aeruginosa the patient was colonized by BURKHOLDERIA species. Do you consider this colonization as a contraindication for lung transplantation?**

**(one choice possible)**

1. No
2. Yes, absolute contraindication
3. Yes, relative contraindication
4. Yes, only if strain is pan-resistant

**Case 3**

**A 63-year-old man will benefit from a lung transplantation in your center for end-stage COPD. In his medical history, he had 10 months ago an acute COPD exacerbation with a WILD Pseudomonas aeruginosa documented in his sputum. The patient was successfully treated with antibiotics. In your pretransplantation assessment, you performed a broncho-alveolar lavage. No more Pseudomonas aeurginosa or another micro-organism was isolated in this lavage.**

**What do you think about the patient colonization?**

**(one choice possible)**

1. You consider no colonization
2. You consider a Pseudomonas aeruginosa colonization
3. other

**You want to carry out an antimicrobial prophylaxis. Which PERIOPERATIVE antimicrobial prophylaxis do you prescribe?**

**(multiple choices possible)**

1. antipseudomonal third-generation cephalosporins (e.g. ceftazidime)
2. fourth-generation cephalosporins (e.g. cefepime)
3. fifth-generation cephalosporins (e.g. ceftaroline)
4. new antipseudomonal cephalosporins with a betalactamase inhibitor: ceftazidime + avibactam
5. new antipseudomonal cephalosporins with a betalactamase inhibitor: ceftolozane + tazobactam
6. piperacillin + tazobactam
7. carbapenem: imipenem
8. carbapenem: meropenem
9. carbapenam: doripenem
10. antibiotics with activity against MRSA pathogens: vancomycin
11. antibiotics with activity against MRSA pathogens: linezolid
12. antibiotics with activity against MRSA pathogens: teicoplanin
13. fluoroquinolone: ciprofloxacin
14. fluoroquinolone: levofloxacin
15. aminoglycoside: amikacin
16. aminoglycoside: tobramycin
17. aminoglycoside: gentamycin
18. colymicin (IV and/or aerosol)
19. other

**In such a case where a bacteria was isolated at least once and never again found on the last samples, which delay between the last bacteria isolation and the lung transplantation you think is sufficient to consider that prophylaxis should not target this bacteria?**

**(one choice possible)**

1. less than 15 dyas
2. 15 days
3. 1 month
4. 3 months
5. 6 months
6. 1 years
7. more than one year
8. other

**If the Pseudomonas aeruginosa isolated in the patient lung samples 10 months ago was a MULTI-DRUG resistant microorganism? Which PERIOPERATIVE antimicrobial prophylaxis do you perform?**

**(multiple choices possible)**

1. You consider no colonization and do the same as Case 1
2. antipseudomonal third-generation cephalosporins (e.g. ceftazidime)
3. fourth-generation cephalosporins (e.g. cefepime)
4. fifth-generation cephalosporins (e.g. ceftaroline)
5. new antipseudomonal cephalosporins with a betalactamase inhibitor: ceftazidime + avibactam
6. new antipseudomonal cephalosporins with a betalactamase inhibitor: ceftolozane + tazobactam
7. piperacillin + tazobactam
8. carbapenem: imipenem
9. carbapenem: meropenem
10. carbapenam: doripenem
11. antibiotics with activity against MRSA pathogens: vancomycin
12. antibiotics with activity against MRSA pathogens: linezolid
13. antibiotics with activity against MRSA pathogens: teicoplanin
14. fluoroquinolone: ciprofloxacin
15. fluoroquinolone: levofloxacin
16. aminoglycoside: amikacin
17. aminoglycoside: tobramycin
18. aminoglycoside: gentamycin
19. colymicin (IV and/or aerosol)
20. other

**In such a case where a MULTI-DRUG RESISTANT bacteria was isolated at least once and never again found on the last samples, which delay between the last bacteria isolation and the lung transplantation do you think is sufficient to consider that prophylaxis should not target this bacteria?**

**(one choice possible)**

1. less than 15 dyas
2. 15 days
3. 1 month
4. 3 months
5. 6 months
6. 1 years
7. more than one year
8. other

**Case 4**

**A 64-year-old woman will benefit from a lung transplantation in your center for end-stage COPD. She has no bronchial colonization in her medical history and no micro-organism was documented in the last broncho-alveolar lavage performed in your pretransplantation assessment.**

**The lung transplantation was successfully performed and the recipient transferred to the intensive care unit and then to the department of respiratory diseases. The pathologist call to inform you to the presence of a localized tuberculoid granuloma with caseous necrosis on the lung explant (left upper lobe).**

**There is no clinical or radiological evidence of active mycobacterial infection in post-transplant. What is your strategy?**

**(multiple choices possible)**

1. nothing specific
2. specific mycobacterial PCR on lung explant tissue
3. sputum samples for culture only
4. sputum samples for culture and mycobacterial PCR
5. broncho-alveolar lavage for culture only
6. broncho-alveolar lavage for culture and mycobacterial PCR
7. one screening for mycobacterium species in the recipient
8. repeated screening for mycobacterium species in the recipient
9. other samples for mycobacterial screening (e.g. urine, stools…)
10. other

**Do you perform specific antibiotic prophylaxis?**

**(one choice possible)**

1. no specific prophylaxis
2. specific treatment for latent tuberculosis
3. specific treatment for latent tuberculosis according to PCR on lung explant tissue only
4. specific treatment for others mycobacterium according to PCR on lung explant tissue only

**Case 5**

**A 23-year-old woman is admitted to the ICU after a lung transplantation following cystic fibrosis. She has been colonized with several strains of Pseudomonas aeruginosa (including MDR strains) and methicillin resistant Staphylococcus aureus (MRSA) for several years and has received intra-venous and aerosolized antibiotics during the last year, mainly cefepime, meropenem, linezolid and nebulized colistin.**

**The last sputum examination obtained 3 weeks before lung transplantation was positive with 2 different strains of Pseudomonas aeruginosa: the first one was only susceptible to ciprofloxacin, amikacin and colistin. The second one had a wild phenotype, susceptible to all anti-pseudomonal betalactams and aminosides and ciprofloxacin. Both donor and receiver lung samples have been performed during the surgery.**

**You choose to use an antimicrobial prophylaxis for the post-operative period. Do you use an anti-MRSA antibiotic prophylaxis?**

**(one choice possible)**

1. No
2. Yes, vancomycin,
3. Yes, linezolid,
4. Yes, teicoplanin
5. Yes, other
6. other

**Which other antibiotic or association do you use?**

**(multiple choices possible)**

1. ciprofloxacin + amikacin
2. ceftriaxone (or cefotaxime or non-antipseudomonal cephalosporin) + ciprofloxacin + amikacin
3. piperacillin + tazobactam + ciprofloxacin + amikacin
4. cefepime + ciprofloxacin + amikacin
5. meropenem (or another carbapenem) + ciprofloxacin + amikacin
6. ciprofloxacin alone
7. amikacine alone
8. A non-antipseudomonal betalactam + ciprofloxacin OR amikacin
9. An antipseudomonal betalactam + ciprofloxacin OR amikacin
10. IV colistin (regardless the other antibiotics)
11. nebulized colistin (regardless the other antibiotics)
12. other

**At day 5 following lung transplantation, you obtain the results of surgical lung samples. The donor samples were sterile, the recipient samples retrieve a MRSA and a wild type Pseudomonas aeruginosa. The patient has been weaned from mechanical ventilation and is now receiving 2l oxygen, has no fever and the chest radiography doesn't present any sign of pneumonia. If you had chosen a broad spectrum antibiotic treatment against Pseudomonas aeruginosa, do you perform a de-escalation or do you conserve the same antibiotic-regimen?**

**(multiple choices possible)**

1. No de-escalation and follow up with the same antibiotic regimen
2. Yes de-escalation with ticarcillin
3. Yes de-escalation with ticarcillin-clavulanate
4. Yes de-escalation with piperacillin
5. Yes de-escalation with piperacillin + tazobactam
6. Yes de-escalation with ceftazidime
7. Yes de-escalation with cefepime
8. Yes de-escalation with meropenem (or other carbapenem)
9. Yes de-escalation with one of the preceding drug associated with an aminoglycoside or ciprofloxacin
10. other

**Do you have the same position concerning de-escalation if the patient was still under mechanical ventilation with FiO2 50% and a chest radiography with bilateral aspecific lung interstitial infiltrate but no fever or hyperleucocytosis?**

**(one choice possible)**

1. Yes
2. No

**What is the duration of the antibiotic treatment for this patient, regardless the antibiotics you choose?**

**(one choice possible)**

1. no prophylaxis
2. 1 day
3. 3 days
4. 7 days
5. 14 days
6. 21 days
7. 28 days
8. until indwelling chest tubes are removed
9. until ICU discharge
10. other

**Thank you for your participation in this survey!**

**In case of publication, please specify the name and contact information of the collaborator of your center.**

First name:

Last name:

Institution:

**Do you have a protocol for the management of antibiotic prophylaxis?**

1. Yes
2. No

**Would you mind transfer the protocol to this adress surveyantibioltx@gmail.com ?**

1. Yes
2. No

**I allow the anonymized analysis of my answers.**

1. Yes
2. No

**If you have any comments feel free.**
